# Narrative review of the systemic inflammatory reaction to cardiac surgery and cardiopulmonary bypass

**DOI:** 10.1111/aor.14171

**Published:** 2022-01-21

**Authors:** Enrico Squiccimarro, Alessandra Stasi, Roberto Lorusso, Domenico Paparella

**Affiliations:** ^1^ Division of Cardiac Surgery Department of Medical and Surgical Sciences University of Foggia Foggia Italy; ^2^ Cardio‐Thoracic Surgery Department, Heart & Vascular Centre Maastricht University Medical Centre Maastricht The Netherlands; ^3^ Department of Emergency and Organ Transplantation University of Bari Bari Italy; ^4^ Cardiovascular Research Institute Maastricht Maastricht The Netherlands; ^5^ Division of Cardiac Surgery Santa Maria Hospital, GVM Care & Research Bari Italy

**Keywords:** cardiac surgery, cardiopulmonary bypass, genomics, inflammatory reaction, transcriptomics

## Abstract

**Background:**

Data from large cardiac surgery registries have been depicting a downward trend of mortality and morbidities in the last 20 years. However, despite decades of medical evolution, cardiac surgery and cardiopulmonary bypass still provoke a systemic inflammatory response, which occasionally leads to worsened outcome. This article seeks to outline the mechanism of the phenomenon.

**Methods:**

A thorough review of the literature has been performed. Criteria for considering studies for this non‐systematic review were as follows: observational and interventional studies investigating the systemic inflammatory response to cardiac surgery, experimental studies describing relevant molecular mechanisms, and essential review studies pertinent to the topic.

**Results:**

The intrinsic variability of the inflammatory response to cardiac surgery, together with its heterogenous perception among clinicians, as well as the arduousness to early discriminate high‐responder patients from those who will not develop a clinically relevant reaction, concurred to hitherto unconclusive randomized controlled trials. Furthermore, peremptory knowledge about the pathophysiology of maladaptive inflammation following heart surgery is still lacking.

**Conclusions:**

Systemic inflammation following cardiac surgery is a frequent entity that occasionally becomes clinically relevant. Specific genomic differences, age, and other preoperative factors influence the magnitude of the response, which elements display extreme redundancy and pleiotropism that the target of a single pathway cannot represent a silver bullet.

## Graphical abstract

The inflammatory reaction to cardiac surgery: predisposition, damage‐associated molecular patterns, and worsened outcome.

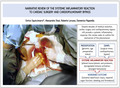

## 1 INTRODUCTION

Cardiac surgery demonstrates non‐negligible rates of mortality and major morbidities. In 1998, the Society of Thoracic Surgeons, which listed more than 170.000 coronary artery bypass grafting (CABG) procedures in the United States, reported 2.6% hospital mortality and 35% of perioperative complications, respectively.^1^ More than 20 years later, the 2021‐update depicts a substantially ameliorated scenario with 1.8% mortality and a decrease in major complications to 11.4% albeit higher‐risk patients have been included in the database over time.^2^

Despite decades of innovation in terms of surgical techniques, cardiopulmonary bypass (CPB), and perioperative care, heart surgery is still associated with adverse events attributed to the high‐risk population, the invasiveness of the practice, and ultimately to CPB that enhances variable degrees of systemic response that is self‐terminating in some circumstances, whereas on occasion plummets toward organ failure and worsened outcome.^3^, ^4^

The only definition of the systemic inflammatory reaction syndrome (SIRS) dates back to 1991 and nowadays merely represents an obsolete and unreliable diagnostic tool for sepsis (Table 1).^5^ While the medical community redefined sepsis as a life‐threatening organ failure secondary to a maladaptive reaction to infection (i.e., pathogen‐associated molecular patterns—PAMPs), the clinical discrimination of SIRS is still controversial, peculiarly in cardiac surgery: a coherent definition would depict a life‐threatening organ dysfunction caused by the dysregulated host‐response to the damage‐associated molecular patterns (DAMPs) associated with surgery and CPB.^6^

**TABLE 1:** SIRS criteria^5^

SIRS as the evidence of two or more of the following criteria	
1	Body temperature >38°C or <36°C
2	Heart rate >90 beats per minute
3	Respiratory rate >20 breaths per minute, or hyperventilation (PaCO_2_ < 4.3 kPa)
4	Alteration in the leukocyte count (>12 000/µl, <4000/µl, or the presence of >10% immature neutrophils)

Given the complexity of this reaction and its multiple and unfathomed aspects, we provide a multifaceted description of the phenomenon by means of a comprehensive overview of the most recently published studies.

## 2 GENOMIC VARIANTS

The non‐recognition of the patients prone to an excessive response represents the *Achilles’ heel* of the randomized controlled trials (RCTs) that failed to demonstrate the advantages by prophylactically addressing inflammation following cardiac surgery.^7^, ^8^, ^9^ In this sense, leukocytosis and a younger age were recently highlighted as variables predictive of a clinically relevant response.^4^, ^10^ Genomic differences might explain the heterogeneity. For instance, gene variants codifying for Interleukin‐6 (IL‐6; ‐572G>C, ‐174G>C) were linked with increased cytokines’ production.^11^, ^12^, ^13^, ^14^ Likewise, a highly prevalent single‐nucleotide polymorphism (SNP) of the IL‐18 gene (9545T>G) was associated with greater IL‐18 and tumor necrosis factor (TNF)‐α production, and a reduced anti‐inflammatory IL‐10 generation.^15^ Some authors also speculated on the possible link between the Apolipoprotein E4 allele and cardiac surgery–related cerebral injury.^16^, ^17^ However, just a positive correlation with increased IL‐8 and TNF‐α levels, together with reduced IL‐10, was reported.^17^, ^18^ The same SNP (wild A allele) was also reported to have a borderline significant association with 30‐day and 5‐year mortality in the INFLACOR study whose post‐hoc analysis interestingly showed the C allele of the ‐572G>C IL‐6 SNP as associated with a significantly reduced effect of prophylactic dexamethasone on postoperative IL‐6 levels compared with the G allele (*p* = 0.0038), whereas its blunting effect upon postoperative C‐reactive protein (CRP) was not genotype dependent.^19^, ^20^ In point of fact, this further corroborates the hypothesis of interindividual genetic differences as responsible for the failure of RCTs and could lead to a “genotype‐suited” dexamethasone prophylaxis in patients experiencing CPB surgery.^8^

Addressing the complexity of the genome‐disease relationships, the second‐generation studies overcame the shortcomings of the candidate‐gene approach obtaining a complete genotype–phenotype evaluation. For instance, the development of the so‐called “*genome‐wide association studies*” allowed the identification of specific genes/intragenic regions’ variants associated with postoperative MI after CABG, with some of these being involved in inflammation and ischemia/reperfusion injury (IRI).^21^ Notwithstanding, postoperative MI represents the most frequent cardiac complication following CABG, and it does not necessarily pertain to systemic inflammation.

## 3 FUNCTIONAL GENOMIC/TRANSCRIPTOMIC and PROTEOMIC

The inflammatory response to CPB has mainly been analyzed by correlating biomarkers with triggers and outcomes. By contrast, other investigations identified gene expression‐profiles by means of a deductive approach, thus characterizing expression‐patterns of genes entailed in inflammation and as associated with clinical outcomes.^22^

In fact, oligonucleotide microarray analyses of 12 625 genes performed on atrial and skeletal muscle samples from patients undergoing CABG clarified specific expression‐patterns in which inflammation and apoptosis played a major role (i.e., c‐FOS, expression ratio (ER) post‐ vs. pre‐CPB 20:1). High upregulation of transcription‐activators and stress‐genes was also reported (e.g., oncogene MYC, ER 5:1), whereas immunoglobulin genes displayed a marked downregulation (e.g., immunoglobulin‐γ‐3, ER 1:47).^23^

Differential regulation of genes in atrial samples from diabetic patients undergoing CABG and matched non‐diabetic ones was also scrutinized revealing different expression‐profiles between the groups with most of the upregulated genes being transcription factors and proinflammatory biomarkers. Among the genes upregulated exclusively in the diabetic cohort, the oncogenes MYC/JUN, and cytokines (e.g., IL‐1β, IL‐8) stood out. Moreover, increased expression of vascular endothelial growth factor (VEGF) was observed.^24^ Higher levels of circulating VEGF in diabetic patients were also reported in another comparison with matched non‐diabetic patients, with the diabetic group experiencing greater weight gain and hospitalization time. In fact, the diabetic group demonstrated augmented mRNA expression of hypoxia‐inducible factor (HIF‐α)—a potent stimulator of VEGF—as well as HIF‐α‐related transcription factors.^25^

CABG with and without CPB underwent similar analysis to assess whether off‐pump CABG could lead to a more benign gene expression‐profile.^26^ Patients experiencing CPB demonstrated augmented expression of leukocyte‐mRNA not only for proinflammatory cytokines, CAMs (i.e., platelet endothelial cellular adhesion molecule—PECAM) but also for IL‐10 and heme oxygenase‐1.^27^ Changes over time at the mRNA level in monocytes of patients who underwent CABG were also evaluated revealing significant upregulation of IL‐6 and IL‐8 genes, peaking at the end of the procedure, and dropping to baseline 24 h postoperatively.^28^ Total‐RNA microarray analysis allowed a reliable comparison between gene expression‐patterns after on‐pump or off‐pump CABG. Remarkably, stress‐related genes upregulated with CPB showed a tendency to a similar but milder expression in off‐pump CABG, characterized by a delayed kinetic, whereas other proinflammatory genes entailed in cell–cell signaling, and apoptosis like CAMs and toll‐like receptors showed exclusive upregulation in the CPB‐group. Proteomic analysis revealed specific cytokines/chemokines, which underwent augmented expression exclusively post‐CPB (e.g., IL‐8, IL‐10, TNF‐α etc) with others (IL‐6 and Interferon‐γ; IFN‐γ) demonstrating just a similar but delayed induction in off‐pump CABG.^29^

Finally, a recent genome‐wide transcriptional analysis on circulating blood cells revealed a gene‐regulatory network consisting of 50 genes involved in a coordinated response to CPB and IRI. Interestingly, this analysis showed a hierarchic structure with hub nodes represented by key sensors of hypoxia and IRI (i.e., HIF‐α), and several downstream pro‐/anti‐inflammatory genes.^22^

The *primum movens* of the inflammatory reaction is the exposure of blood to foreign materials. Interplays between free ions and ionic sites on the surfaces implicate local alterations of the water structure. Furthermore, plasma proteins and leukocytes undergo adsorption by the system enduring conformational changes and receptors exposition.^3^ Hence, a dynamic equilibrium of continuous peptides adsorption/release with different grades of denaturation is established. This unphysiological condition conveys to an uncharted activation of a complex system involving plasma proteins, transcription factors, molecular complexes, and cellular elements.

### 3.1 Complement

Alternative cascade activation occurs spontaneously via hydrolysis of C3 and its adsorption onto the CPB surfaces. Clinical studies reported either the direct biding of C1q and mannose‐binding lectin (MBL) to the surfaces, or their indirect binding though host‐antibodies and CRP,^30^, ^31^ ergo suggesting that artificial materials represent a major determinant of early complement activation. In vitro studies described MBL, ficolin‐1, ‐2, and ‐3 deposits on the surface of uncoated polyurethane tubing as well as C4 activation products.^32^ In this sense, it was reported that albumin–heparin coating outperforms phosphorylcholine on plasma depletion of ficolin‐2, a pattern‐recognition molecule of the lectin pathway.^33^

Furthermore, protamine administration prompts the formation of complexes that enhance the classical pathway (no activation of C2/C4 is observed in patients undergoing off‐pump CABG without resort to protamine).^34^

The clinical relevance of complement is demonstrated by the association of Cd4‐CRP complexes with arrhythmias after CABG. Cd4‐CRP was described as marker of delayed (5 days) complement activation (i.e., biphasic complement‐response).^34^ The magnitude of complement activation and its impact on the outcome has not been clearly outlined, it is rather heterogeneous and may be influenced by variation of certain genes.^35^ The combined MBL2 5′ LYQA secretor haplotype (CGTCGG) and 3′ haplotype (CGGGT) presented increased risk of MI following CABG.^36^ On the contrary, MBL2 A/O and O/O genotypes appear as protective factor, whereas MBL2 YA/YA genotypes were associated with maladaptive inflammation.^37^

### 3.2 Cytokines

The mechanism of postoperative inflammation involves a plethora of cytokines, which are pleiotropic (single cytokine—several functions) and redundant (multiple cytokines—same effect).^3^

DAMPs/PAMPs promote the production of IL‐1 that is made up of two structurally interconnected molecules: IL‐1α and IL‐1β. IL‐1 mediates various aspects of inflammation like fever, mediator release, endothelial impairment, and vasoplegia. DAMPs/PAMPs also promote the synthesis of TNF, which is a major contributor to inflammation enhancing the production of hepatic proteins and activating polymorphonuclear neutrophils (PMNs).^38^ Furthermore, the release of TNF‐α during cardiac procedures provokes renal infiltration and fibrin accumulation.^39^ IL‐6 plays a dominating role in the modulation of inflammation following cardiac surgery throughout functions superimposable of the above discussed. Besides, IL‐6 acts on hypothalamic–pituitary–adrenal axis and directly on adrenal cells, leading to the production of corticosteroids and adrenocorticotropic hormone, thus showing a paradoxical counter‐regulatory effect. IL‐8 was the first recognized neutrophil‐specific chemotactic agent via bridging ECs and neutrophils while acting as carrier through the endothelium. PMN degranulation and production of reactive oxygen species (ROS) was also attributed to IL‐8. Finally, IL‐10 is an anti‐inflammatory cytokine that suppresses nuclear factor‐kB (NF‐kB) and compromises its downstream target genes expression.^40^

**FIGURE 1:**
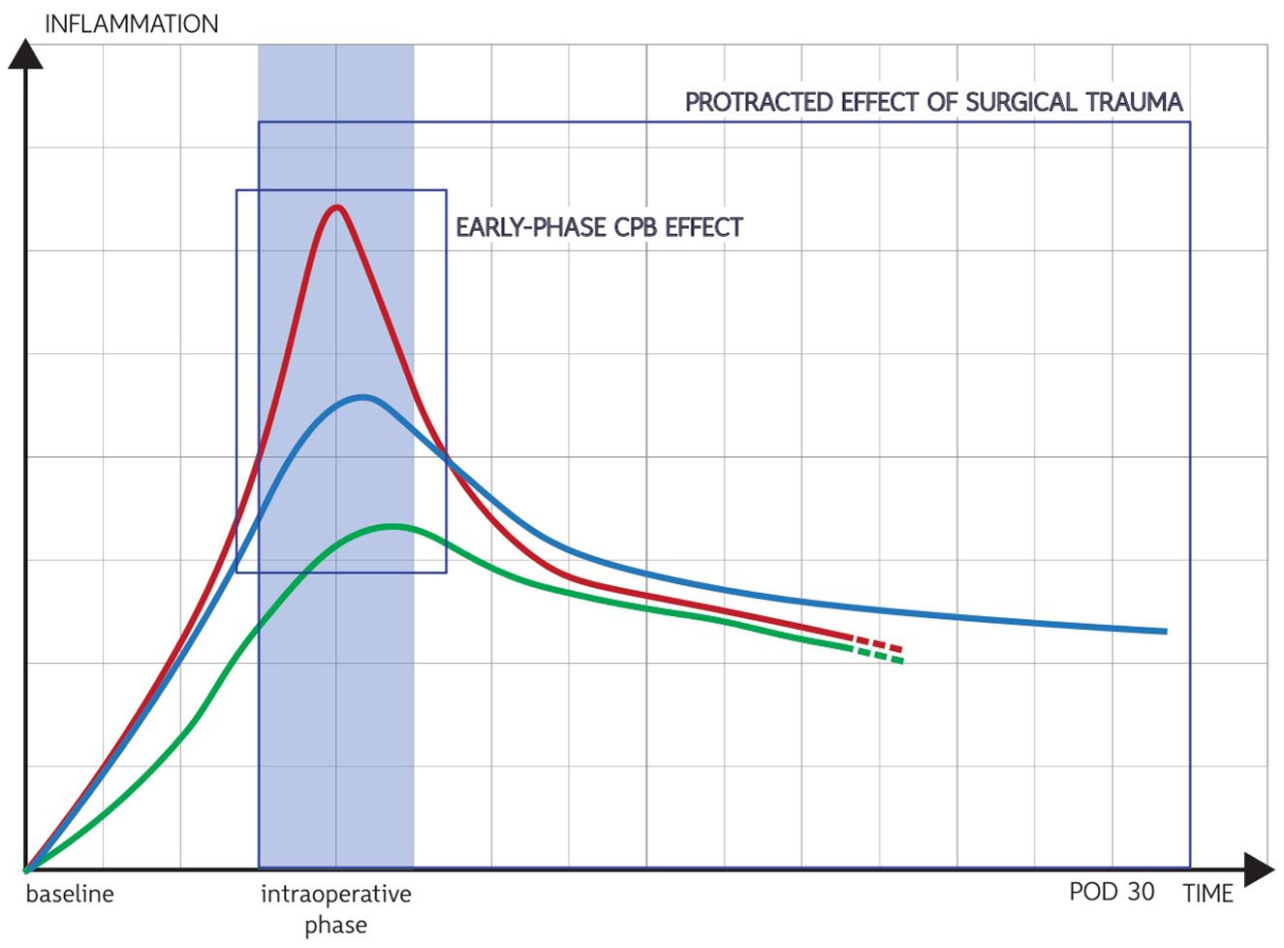
Inflammation “kinetic” in on‐pump and off‐pump coronary bypass surgery.^41^ Red line: TNF‐ α, IL‐8, IL‐10 (on‐pump group); green line: TNF‐ α, IL‐8, IL‐10 (off‐pump group); and blue line: IL‐6 protracted elevation both in on‐pump and off‐pump [Color figure can be viewed at wileyonlinelibrary.com]

A modern and intriguing manner to characterize the systemic inflammatory reaction to cardiac surgery and CPB would consist in the ascertainment of the relative weight of these distinct triggers. Indeed, evidence of different inflammatory mediators’ signature profiles has been provided via the direct comparison of case series either with or without the resort to CPB.^41^ More specifically, some cytokines among which TNF‐α, IL‐8, and IL‐10 accrue both after on‐pump and off‐pump CABG and, despite peaking earlier and higher in on‐pump procedures, they progressively and prematurely fade to baseline in both cohorts.^41^ Conversely, other cytokines’ kinetic (e.g., IL‐1 and IL‐6) do not provide evidence of differential activation between on‐pump and off‐pump CABG procedures. Hence, it can be speculated that the role of CPB in the onset and subsistence of SIRS is very limited to early postoperative phase, whereas the surgical trauma is likely to represent the major determinant in the late phase (Figure 1). The time‐span of this late phase also underwent investigation showing a protracted period of inflammation with IL‐6 and high‐sensitivity CRP levels not decreasing to baseline even after 30 days.^42^ This enduring increase in inflammatory cytokines (i.e., as well as the biphasic activation of the complement system^34^) might play a role in the risk of postoperative cardiovascular events.

Perhaps, the identification of cytokine hubs would possibly reframe our understanding of the inflammatory response to cardiac surgery and CPB and expand the treatment possibilities. However, translating this molecular‐based model, which is nowadays pioneered in areas such as the immune‐mediated inflammatory diseases (e.g., rheumatoid arthritis, Crohn's disease, etc) to postoperative SIRS would sound arduous since the development of cytokine hubs relied on decades of investigations upon the efficacy of drug therapies (i.e., monoclonal antibodies) each targeting very specific cytokines, and more importantly postoperative SIRS is not in itself a disease, it is rather a syndrome encompassing numerous triggers and pathway activation.^43^

### 3.3 Coagulation and fibrinolysis

Postoperative inflammation integrates coagulation and fibrinolysis eventually leading to bleedings and thrombosis. Blood exposure to artificial materials initiates intrinsic coagulation through factor XIIa that activates factor XI. The cascade continues with the activation of factor X: the convergence of intrinsic/extrinsic coagulation that forms the prothrombinase complex needed to catalyze the conversion of prothrombin to thrombin. Thrombin converts fibrinogen in fibrin which in turn activates platelets and stimulates ECs to generate von Willebrand factor. Thrombin is a formidable DAMP triggering the production of vasoactive agents and chemoattractants and determining neutrophil adhesion and endothelial impairment.^44^

Tissue factor (TF) is a transmembrane glycoprotein expressed by subendothelial cells (notwithstanding, soluble TF‐microparticles were identified).^45^ The TF‐factor VII complex promotes factor X activation. Further leukocyte TF‐exposition was also observed leading to massive factor VII activation.^45^ Finally, coagulation is fully set in motion leading to consumptive coagulopathy, bleedings, and thrombosis.

Fibrin networks are later removed by fibrinolysis that is hereafter suppressed. Its central reaction is the activation of plasminogen in plasmin, which cleaves fibrin generating breakdown products like d‐dimers. The urokinase plasminogen activator (u‐PA) plays less of a role compared with tissue plasminogen activator (t‐PA), which makes hemostasis to occurs in a continuous‐loop within the pericardial wound and CPB.^46^ The overproduction of t‐PA is also ascribable to the stimulating effect of thrombin on ECs and to the activity of factor XIIa and kallikrein.^34^

### 3.4 NF‐kB

NF‐kB epitomizes a class of five inducible‐transcription factors having different DNA targets and cell specificity, all involved in the regulation of genes implicated in the immuno‐inflammatory response. It is normally sequestered in the cytoplasm of all nucleated cells until activation by DAMPs/PAMPs.

NF‐kB binds a group of inhibitory proteins, called I‐kBs, containing a repeated ankyrin‐sequence that veils nuclear localization‐signals. When stimulated, I‐kB kinases phosphorylate the NF‐kB‐IkB complex ergo NF‐kB moves to the nucleus where it binds specific DNA/kB‐enhancers. The correlation of NF‐kB with ischemia is demonstrated by the abrupt reduction of cytoplasmic‐IkB and increased nuclear NF‐kB p65 subunit expression following ischemia.^47^ Aside of the IL‐10 protective effect (i.e., I‐kB phosphorylation suppression/inhibition of NF‐kB‐DNA coupling^40^), repression of NF‐kB activity and consequent amelioration of IRI were obtained by annexin A1 (ANX‐A1; i.e., endogenous anti‐inflammatory protein associated with I‐kB kinases binding to Heat Shock Protein Family B Member 1) peptidomimetic Ac2‐26 in CPB animal models.^48^

NF‐kB activation occurs also throughout the RANKL–RANK interaction: the receptor activator of the NF‐kB ligand (RANKL), its membrane receptor RANK and its decoy receptor osteo‐protegrin (OPG) have been described as involved in inflammatory diseases.^49^ Interestingly, evidence of OPG/RANKL/RANK activation following either on‐pump or off‐pump CABG was reported: augmented expression of NF‐kB p50 subunit in T‐cells was associated to increased postoperative OPG levels.^50^

However, controversial evidence was provided about the role of NF‐kB during acute hypoxia and IRI: some studies even suggest a cardio‐protective action in contrast to some other investigations that ascertained the elicitation of downstream inflammatory mediators.^51^

### 3.5 Inflammasomes

With a prefix referring to inflammation and a suffix deriving from the Greek “soma” (“body”—complex), the inflammasome was originally described in 2002 as an inducible protein complex (structurally/functionally similar to the better‐known apoptosome) whose assembly prompts inflammation.^52^ NLRP3 is characterized by leucine‐rich repeat domains, a nucleotide‐binding domain, and an N‐terminal pyrin domain. NOD‐like receptors (NLRs) can sense DAMPs, thus, trigger the formation inflammasomes that activate caspase‐1 via the adaptor protein ASC, producing cytokines and inducing pyroptosis: lysis characterized by membrane pores that annihilate the ionic gradient, leading to swelling and degranulation.^53^ However, the process of cytokines generation and secretion is controversial: all models proposed (e.g., exosomes, microvesicles, pyroptosis, etc) led to evidence both for and against each hypothesis.^54^ The comprehension of inflammasome biology is increasing each day, but still, it is embryonic, particularly in cardiac surgery.

## 4 CELLULAR ELEMENTS

### 4.1 Endothelial cells

Albeit its heterogeneity (different morphology/function in different organs) the endothelium is a dynamic modulator balancing between pro‐ and anti‐inflammatory/coagulative conditions. Activated ECs expose CAMs and synthetize chemotactic substances while leukocytes expose high‐affinity ligands, which bind ECs leading to the “rolling” that further injuries the endothelium.

CPB‐driven flow implicates shear‐stress modifications and upregulate shear‐stress‐responsive genes.^55^ Furthermore, ECs modulate vasomotion via endothelium‐derived relaxing (e.g., NO, prostacyclin, bradykinin) and contracting factors (e.g., endothelin‐1, angiotensin II, thromboxane A2) leading to macro‐/micro‐circulatory disturbances.^56^ Shear‐stress induces iNOS activity and chemokine repression, conversely the continuous flow reduces NO production. Circulating cytokines further inhibit NO generation causing the so‐called “endothelial stunning”.^57^ The endothelial proinflammatory‐phenotype links inflammation and hemostasis feeding back on platelets, leukocytes, and further EC activation.

### 4.2 Glycocalyx

The vascular intima is a gargantuan surface first regarded as a mere blood tissues’ physical barrier. The existence of a coating was only proposed half‐century later, and 25 years more were needed to visualize this irregular, fluffy film extending into the vascular lumen.^58^

The glycocalyx is indeed a negatively charged gel‐like coating composed of membrane‐bound proteoglycans, glycoproteins, glycosaminoglycans (GAGs), adherent and soluble plasma proteins. Its structure/composition is greatly variable given the perpetual turn‐over with the circulation.^59^ Proteoglycans, syndecans, and glypican‐1 core proteins, are anchored to ECs. Several negatively charged GAGs covalently bound to proteoglycans (e.g., heparan sulfates, chondroitin sulfates) enable electrostatic interplays with circulating albumin, antithrombin, and fibrinogen.^60^

The glycocalyx is such a fundamental regulator of the endothelial mechanotransduction that the Starling equation was updated to include its contribution.^61^ Moreover, it has several other functions like managing vascular permeability and inhibiting thrombosis.^59^

Glycocalyx degradation contributes to microcirculatory dysfunction.^62^ Inflammation is a precipitating factor for its integrity, which decays right after CPB‐onset.^63^ Indeed, evidence of a clinically relevant association between glycocalyx shedding, augmented inflammation, and postoperative vasoplegia was provided.^64^, ^65^

### 4.3 CAMs

The first leukocyte‐endothelium connection consists in a loose attachment mediated by three glycoproteins named selectins. L‐selectin is the smallest of the vascular selectins expressed on leukocytes. It binds ECs’ transmembrane CD34. Historically, many anti‐inflammatory artifices uselessly endeavored to reduce its expression without any compelling effectiveness demonstration (e.g., cooling, leukocyte filters, coatings).^66^ E‐selectin is transiently expressed on activated ECs (transcribed and transported to the membrane following DAMP stimulation). Despite of its specificity, several investigations failed to record significant increases of soluble E‐selectin during CPB, whereas E‐selectin‐mRNA induction has been reported.^67^ P‐selectin is found in ECs and platelets (respectively stored in Weibel–Palade bodies and α‐granules). Following DAMP stimulation (as demonstrated by P‐selectin‐mRNA upregulation), it reaches the membrane and corroborates its binding not only to the P‐selectin glycoprotein ligand‐1 expressed on leukocytes but also to heparan sulfates and fucoidans.^68^

Integrins encompass the largest array of CAMs. They are obligate heterodimers composed by noncovalently associated subunits each penetrating the membrane and having cytoplasmic domains. Integrins secures leukocyte adhesion to ECs. The structural/functional integrins classification is based on their β‐chain. β2‐integrins are the most extensively studied integrins in heart surgery. Macrophage‐1 antigens (MAC‐1) and lymphocyte function–associated antigens‐1 (LFA‐1) play a part in inflammation modulating neutrophil adhesion and transmigration.^69^ β1‐integrins, known as very late antigen (VLA)‐integrins, are also widely expressed. VLA‐4 is a major contributor to leukocyte‐ECs interplay by means of its binding with the endothelial vascular cell adhesion molecule (VCAM)‐1 ligand and its ability to settle selectin‐independent leukocyte recruitment.^69^

The *immunoglobulin superfamily (IgSF)* are CAMs characterized by extracellular Ig‐like domains, mainly expressed on ECs (whereas soluble IgSF‐CAMs also exist). Following DAMP stimulation, augmented generation of ICAM, VCAM, and PECAM occurs. ICAM‐1 is continuously expressed on ECs and leukocytes acting as ligand for β2‐integrins. Hence, leukocytes bind to ECs through ICAM‐1 to LFA‐1/MAC‐1 bridges, then extravasate.^70^ VCAM‐1 is a sialo‐glycoprotein expressed on ECs that primarily binds VLA‐4. The attachment of ICAMs/VCAMs with integrins leads to firm ECs‐leukocytes adhesion, extravasation, and granules content release. Leukocyte's diapedesis is facilitated by PECAM‐1 that decreases neutrophils adhesion to ICAM‐1 finalizing their trans‐endothelial migration. Hence, PMNs undertake chemotaxis toward inflammation loci where oxygen‐dependent/independent mechanisms occur. The second consist in granules cytotoxic content release. Furthermore, PMNs’ NADPH‐oxidase is the main source of ROS, which generation is triggered by DAMPs and was termed “*respiratory burst*.” A dysregulated ROS production (occurring up to 48 h after CPB) leads to vascular/tissue damage resulting in organ dysfunction.^71^

### 4.4 Platelets

Contemporary knowledge entrusts a crucial purpose to platelets beyond hemostasis: the thread of thrombosis and immuno‐inflammation.^72^ The blood–surface interaction primes thrombocytes determining a dynamic equilibrium of adsorption‐release of degraded platelets.^3^ Relevant hemodilution and mechanical disruption of thrombocytes are other factors that sum up to the consumptive coagulopathy within the mediastinal‐wound and concur to thrombocytopenia, thus jeopardizing the outcome (i.e., augmented mortality, strokes, acute kidney injury, infections, and longer in‐hospital/ICU‐stay).^73^

The direct platelet–leukocyte crosstalk is paramount by means of reciprocal activation that determines major phenomena like the multistep pathway of neutrophil extravasation, and the release of NETs, which signaling involves high‐mobility group box 1 that mobilizes NF‐kB via receptors for advanced glycation endproducts^74^ leading to widespread inflammation, and IRI.^3^ In fact, new insights about perivascular mast cell activation via PAF from gut microvascular endothelial‐bound platelets following CPB start to emerge.^75^

Ordinary CPB‐associated DAMPs upregulate platelet‐NLRP3, whereas leukocyte‐NLRP3 activation appears to be independently boosted by platelets in vivo.^76^, ^77^ This mechanism prompted a robust cytokines release, which was faded by platelet depletion.^77^

Platelets even interact with other pathways involved in postoperative inflammation. For instance, complement C5b‐9 has been detected on circulating platelet microparticles.^78^ Platelet microparticles present heterogenous compositions (e.g., cytokines, CAMs, miRNAs, TF). Microparticle‐associated‐TF‐activating extrinsic coagulation, indeed, represents another link between hemostasis and immuno‐inflammation.^79^ DAMP stimulation promotes the production of miRNAs: non‐coding RNAs involved in a not‐fully‐understood genetic material transfer to recipient cells.^80^ Plasma exosomal miR‐223 increases with CPB‐onset and was reported to mitigate IL‐6 and NLRP3 expression in monocytes, thus controversially downregulating inflammation.^81^

CPB‐related DAMPs influence intraplatelet mRNAs ergo the proteome: platelets’ protein‐expression impairments are associated to enhanced Bax apoptotic signaling in cardiac patients.^82^, ^83^ Enhanced apoptosis‐associated platelet dysfunction was reported also in patients who underwent off‐pump CABG, but to a milder extent.^83^

## 5 CONCLUSIONS

The inflammatory response to cardiac surgery is a clinically relevant phenomenon that on occasion worsens patient's outcome.^4^ Its elements are pleiotropic, redundant, and interwoven, representing a non‐linear dynamic system variable, interconnected, and cross‐regulated whose perioperative time‐span is still unclear. Anti‐inflammatory therapies targeting single pathways are unlikely to represent a silver bullet. Interindividual differences in terms of susceptibility to the DAMPs and, to a lesser extent to the PAMPs, associated with cardiac procedures explain the lack of universal efficacy of several anti‐inflammatory strategies.

Perhaps, the identification of cytokine hubs pertinent to specific SIRS‐related complications or, more likely, the recognition of dynamic codependent cytokine hubs would point to molecular pathotypes, thus pave the way for a reshaped understanding of postoperative SIRS, and finally for effective targeted interventions.

## CONFLICT OF INTEREST

None declared.

## AUTHOR CONTRIBUTIONS

Conceptualization, design, writing, and editing of the work by Enrico Squiccimarro, Alessandra Stasi contributed to the drafting of the work and revised the manuscript. Roberto Lorusso and Domenico Paparella contributed to the conceptualization of the work, and by critical revisions and draft editing.
